# Feasibility of a multiparametric MRI protocol for imaging biomarkers associated with neoadjuvant radiotherapy for soft tissue sarcoma

**DOI:** 10.1259/bjro.20200061

**Published:** 2021-01-15

**Authors:** Lucy Kershaw, Laura Forker, Darren Roberts, Benjamin Sanderson, Patrick Shenjere, James Wylie, Catherine Coyle, Rohit Kochhar, Prakash Manoharan, Ananaya Choudhury

**Affiliations:** ^1^ The University of Manchester, Manchester Academic Health Science Centre, The Christie NHSFT, Manchester, United Kingdom; ^2^ Translational Radiobiology Group, Division of Cancer Sciences, The University of Manchester, Manchester Academic Health Science Centre, The Christie NHSFT, Manchester, United Kingdom; ^3^ Dept of Histopathology, The Christie NHSFT, Manchester, United Kingdom; ^4^ Dept of Clinical Oncology, The Christie NHSFT, Manchester, United Kingdom; ^5^ Dept of Radiology, The Christie NHSFT, Manchester, United Kingdom

## Abstract

**Objective:**

Soft tissue sarcoma (STS) is a rare malignancy with a 5 year overall survival rate of 55%. Neoadjuvant radiotherapy is commonly used in preparation for surgery, but methods to assess early response are lacking despite pathological response at surgery being predictive of overall survival, local recurrence and distant metastasis. Multiparametric MR imaging (mpMRI) is used to assess response in a variety of tumours but lacks a robust, standardised method. The overall aim of this study was to develop a feasible imaging protocol to identify imaging biomarkers for further investigation.

**Methods:**

15 patients with biopsy-confirmed STS suitable for pre-operative radiotherapy and radical surgery were imaged throughout treatment. The mpMRI protocol included anatomical, diffusion-weighted and dynamic contrast-enhanced imaging, giving estimates of apparent diffusion coefficient (ADC) and the area under the enhancement curve at 60 s (iAUC_60_). Histological analysis of resected tumours included detection of CD31, Ki67, hypoxia inducible factor and calculation of a hypoxia score.

**Results:**

There was a significant reduction in T1 at visit 2 and in ADC at visit 3. Significant associations were found between hypoxia and pre-treatment iAUC_60_, pre-treatment ADC and mid-treatment iAUC_60_. There was also statistically significant association between mid-treatment ADC and Ki67.

**Conclusion:**

This work showed that mpMRI throughout treatment is feasible in patients with STS having neoadjuvant radiotherapy. The relationships between imaging parameters, tissue biomarkers and clinical outcomes warrant further investigation.

**Advances in knowledge:**

mpMRI-based biomarkers have good correlation with STS tumour biology and are potentially of use for evaluation of radiotherapy response.

## Introduction

Soft tissue sarcoma (STS) is a rare and heterogeneous malignancy. There were 3298 new diagnoses in the UK in 2010, and the 5 year overall survival rate was 55%.^1^ Tumour grade is prognostic, with 5 year metastasis-free survival rates of 71 and 44% for intermediate and high grade tumours respectively.^2^ Surgery, the main treatment for STS of the limb and trunk, involves excising the lesion along with a tumour-free margin of around 1–2 cm, subject to anatomical constraints. In intermediate/high grade tumours or those which require extensive surgery, this is often not possible without risk to function. In these situations, radiotherapy is employed either pre- or post-operatively. Post-operative radiotherapy requires a higher dose being delivered to a larger volume but can be reserved for situations where an inadequate margin is obtained. Pre-operative radiotherapy may be used to shrink a tumour, for some subtypes^3^ making a tumour more operable,^4^ or to sterilise the margins in preparation for excision.^5^ The use of a lower dose and reduction in the irradiated volume for pre-operative compared to post-operative radiotherapy results in similar local control, but with a reduction in late toxicity and better function.^6^ There is no consensus on the optimal approach for radiotherapy in STS, with a rationale for both pre- and post-surgical radiotherapy.

There is currently no method to assess early response to pre-operative radiotherapy, despite pathological response at surgery being predictive of overall survival, local recurrence^7^ and distant metastasis.^8,9^ Imaging biomarkers are attractive because they are non-invasive, and MR is available in most centres. Volume change in STS during pre-operative radiotherapy is minimal despite marked pathological response,^3^ and tumour size changes (RECIST criteria and/or three-dimensional tumour volumes) are poor predictors of tumour-free surgical margins, local control^4^ and overall survival.^10^

Multiparametric MR imaging (mpMRI) has been used to demonstrate radiotherapy response early in treatment, which might allow prompt progression to definitive treatment in poorly responding tumours.^11–13^ Conversely, some STS such as myxoid liposarcoma may respond adequately with lower doses of radiotherapy reducing late effects without compromising outcome.^14^ Use of functional measures such as dynamic contrast-enhanced (DCE) MRI (measuring tissue microvasculature), and diffusion-weighted imaging (DWI) (measuring restriction of water molecule diffusion) has been shown to increase the ability of imaging to reflect the amount of tumour necrosis,^15–18^ but protocols varied widely and image analysis often required careful selection of tumour subregions. Delivery of a robust protocol suitable for large multicentre studies is challenging. This study aligns with domain I of the imaging biomarker road map,^19^ assessing the feasibility of delivering a robust protocol across different anatomical sites, but within a single institute.

The overall aim of this study was to identify imaging parameters suitable for investigation as prognostic factors in a larger study. The specific aims were to: (i) develop a well-tolerated imaging protocol allowing for repeat scanning, (ii) identify imaging parameters that change significantly during radiotherapy, and (iii) determine relationships between imaging parameters and histological features in resected tumour tissue. As part of this exploratory assessment, particular attention was focussed on post-surgical hypoxic regions in the resected tumour, a parameter known to affect survival.^20,21^

## Methods and materials

### Patients

In this prospective study, 15 patients with biopsy-confirmed intermediate or high-grade STS suitable for pre-operative radiotherapy (50 Gy in 25 fractions) and radical surgery were recruited. This study had a favourable ethical opinion (13/NW/0500) and all patients gave written informed consent. Patient characteristics are summarised in Table 1.

**Table 1.** Patient characteristics

Patient	Age at first scan	M/F	Tumour location	Tumour type, grade, stage	Status as of Jan 2019
1	73	M	Upper arm	Undifferentiated spindle cell G2, T2bN0M0	No disease
2	56	M	Trunk	Myxoid liposarcoma G3, T2bN0M0	No disease
3	79	M	Upper arm	Myxofibrosarcoma G2, T1bN0M0	No disease
4	27	M	Knee	Myxoid liposarcoma G3, T2bN0M0	No disease
5	29	M	Lower leg	Myxoid chondrosarcoma G3, T2bN0M0	No disease
6	69	F	Trunk	Undifferentiated pleomorphic sarcoma G3, T1aN0M0	No disease
7	41	M	Lower leg	Myxofibrosarcoma G3, T2bN0M0	No disease but chronic inflammation post-surgery
8	62	M	Forearm	Myxofibrosarcoma G3, T1bN0M0	No disease
9	24	M	Knee	Synovial sarcoma G3, T2bN0M0	Lung metastasis resected June 2018, now no disease
10	67	M	Knee	Undifferentiated pleomorphic sarcoma G3, T2bN0M0	Died (Acute Myeloid Leukaemia) Feb 2018
11	33	M	Thigh	Myxoid liposarcoma G3, T2bN0M0	Single metastasis in spine August 2018
12	74	M	Trunk	Undifferentiated spindle cell sarcoma G3, T2bN0M0	No disease

Patients underwent MRI before radiotherapy (within 4 weeks of the start of treatment), in week 2 or 3 of radiotherapy, and 2–4 weeks after radiotherapy. Within 1 month of the post-radiotherapy visit, the tumours were surgically resected. At surgery, proximal, distal, medial, lateral, depth and superficial margins were demarcated.

### Imaging protocol

Patients were imaged at 1.5 T (Siemens MAGNETOM Avanto, Siemens Healthineers, Erlangen, Germany) using either the peripheral or body matrix coil with the appropriate elements of the spine array. Imaging began with standard clinical sequences (pre-contrast transverse and coronal *T*_1_W and *T*_2_W, post-contrast transverse and coronal *T*_1_W with fat saturation, all turbo spin echo) and continued with trial sequences as shown in Table 2. During the dynamic sequence, 0.1 ml/kg Gadovist was injected using a power injector at 2 ml s^−1^, followed by 20 ml saline at the same rate.

**Table 2.** Trial imaging protocol

Sequence	Purpose	Flip angle, TE, TR / ms	Parallel imaging factor	Other	Matrix^a^	FOV / cm
TSE 2D Turbo spin-echo	High resolution *T*_2_W for tumour outlining	150**°** 96, 3890	None	ETL 13	256 × 256 x 20	Limb: 25 × 25 x 10 Trunk: 38.6 × 38.6 x 10
EPI 2D Echoplanar imaging	Diffusion-weighted imaging	- 103, 1,2100	2 AP	EPI factor 128, *B* = 0, 50, 100, 150, 200, 500, 1000 s/mm^2^	128 × 128 x 20	
SRTFE 3D Saturation-recovery turbo field echo	T1 measurement	12**°** 1.52 64, 142, 292, 1050, 2550, 3950	2 AP	TI = 37, 100, 250, 1000, 2500, 3900 ms		
VIBE 3D Volume-interpolated breath-hold imaging	Dynamic contrast-enhanced imaging	16**°** 0.81, 2.63	2 AP	Temporal resolution 1.75 s, 150 dynamics		

TE, echo time; TR, repetition time; TI, inversion time; FOV, field of view; 2D, two-dimensional; 3D, three-dimensional.

a In one case, 26 slices were needed to cover the tumour, leading to a dynamic temporal resolution of 3.2 s, and TR values for the SRTFE of 73, 145, 306, 1060, 2560, and 3960 ms.

### Image analysis

ADC maps were calculated at the time of acquisition. Images were analysed using Python (v. 3.6). T1 maps were calculated by fitting the saturation recovery turbo FLASH equation on a pixel-by-pixel basis.^22^ The integrated area under the curve in the first 60 s after injection (iAUC_60_) was calculated on a pixel-by-pixel basis using trapezoidal integration, after converting the signal intensity *vs* time curves to contrast agent concentration *vs* time curves.^23^

Tumours were outlined on *T*_2_W images by BS (confirmed by a consultant radiologist with expertise in MRI), and volumes automatically calculated from the known voxel size. These regions of interest (ROIs) were transferred to the dynamic images by nearest-neighbour interpolation and to the ADC maps by referring to anatomical landmarks to ensure that the tumour was correctly outlined even in the presence of distortion. ROIs were eroded in-plane by one pixel to avoid partial volume effects at the region edges. The median and interquartile range over the whole tumour ROI was calculated for ADC, iAUC_60_ and T1.

### Histological analysis of resected tumours

Immunohistochemistry was performed on 4 µm sections from formalin-fixed paraffin-embedded (FFPE) tumour resection samples to score hypoxia inducible factor-1 α (HIF-1α), carbonic anhydrase IX (CAIX), antigen KI-67 (Ki67) and CD31. HIF-1α, CAIX and Ki67 staining was performed using the Bond-Max Automated staining system (Leica Biosystems, Milton Keynes, UK). Slides were de-waxed and rehydrated and antigen retrieval was carried out at pH 9.0 for 40  min at 100°C. The primary antibodies were HIF-1a (BD Biosciences 610959; 1:100 dilution), CAIX (NCL-L-CAIX, Novascastra, Leica Biosystems; 1:100 dilution), Ki67 (clone MIB-1, Dako M7240; 1:100 dilution), and CD31 (M0823 Dako; 1:50 dilution). For HIF-1α, Ki67 and CD31 the negative control was mouse IgG1 (Dako X0931) and for CAIX was mouse IgG2a (Dako, Ely, UK, X0943). All dilutions were in antibody diluent (Leica AR9352) and negative controls were diluted to the same protein concentration as the primary. Slides were stained using a standard BOND processing protocol (available on request) and Bond Polymer Refine Detection System (Leica DS9800). Colorectal cancer cell line spheroids with a diameter <500 µm were used as a positive control for HIF-1α and CAIX. A FFPE biopsy of normal human placenta was used as a positive control for CD31.

The percentage of tumour cells per core expressing membranous CAIX was scored by a sarcoma pathologist (PS) at ×8 magnification with negative controls available for comparison. Other markers were scored using automated image analysis (Definiens tissue studio v. 4.2; Definiens, Munich, Germany).

The percentage of tumour material was assessed by a sarcoma pathologist (PS) on a separate H&E stained section. RNA from three 10 µm sections was extracted using the FFPE RNA/DNA Purification Plus Kit (Norgen, Thorold, Ontario, Canada) including DNase I treatment. The High-Capacity cDNA Reverse Transcription Kit (Life Technologies, Paisley, UK) was used to reverse transcribe total RNA. cDNA was preamplified using a custom pool of TaqMan assays (Life Technologies) and TaqMan PreAmp Master Mix (Life Technologies).

Expression of a 24-gene hypoxia signature derived for STS^24^ and 2 endogenous control genes selected for STS^25^ was determined using custom 384-well TaqMan array cards (Life Technologies) on a QuantStudio 12K Flex Real-Time PCR System (Life Technologies) using TaqMan Fast Advanced Master Mix (Life Technologies) according to the manufacturer’s guidelines. The geometric mean of the endogenous control genes was used for normalisation. Hypoxia scores (HS) were calculated as the normalised median expression of the 24 hypoxia genes (note that the median is used due to the small sample size in this study, rather than the method presented previously).^24^

### Statistical analysis

In this small feasibility study, changes in median T1, ADC and iAUC_60_ between the three visits were assessed using a Wilcoxon signed ranks test. Correlations between imaging and histology parameters were assessed by calculating the Pearson correlation coefficient and its associated *p*-value using the *t* distribution. Differences in imaging parameters between the tumours with and without CAIX staining was assessed using the Mann–Whitney *U* test. No correction was made for multiple comparisons.

## Results

### Patients

**Figure 1.**
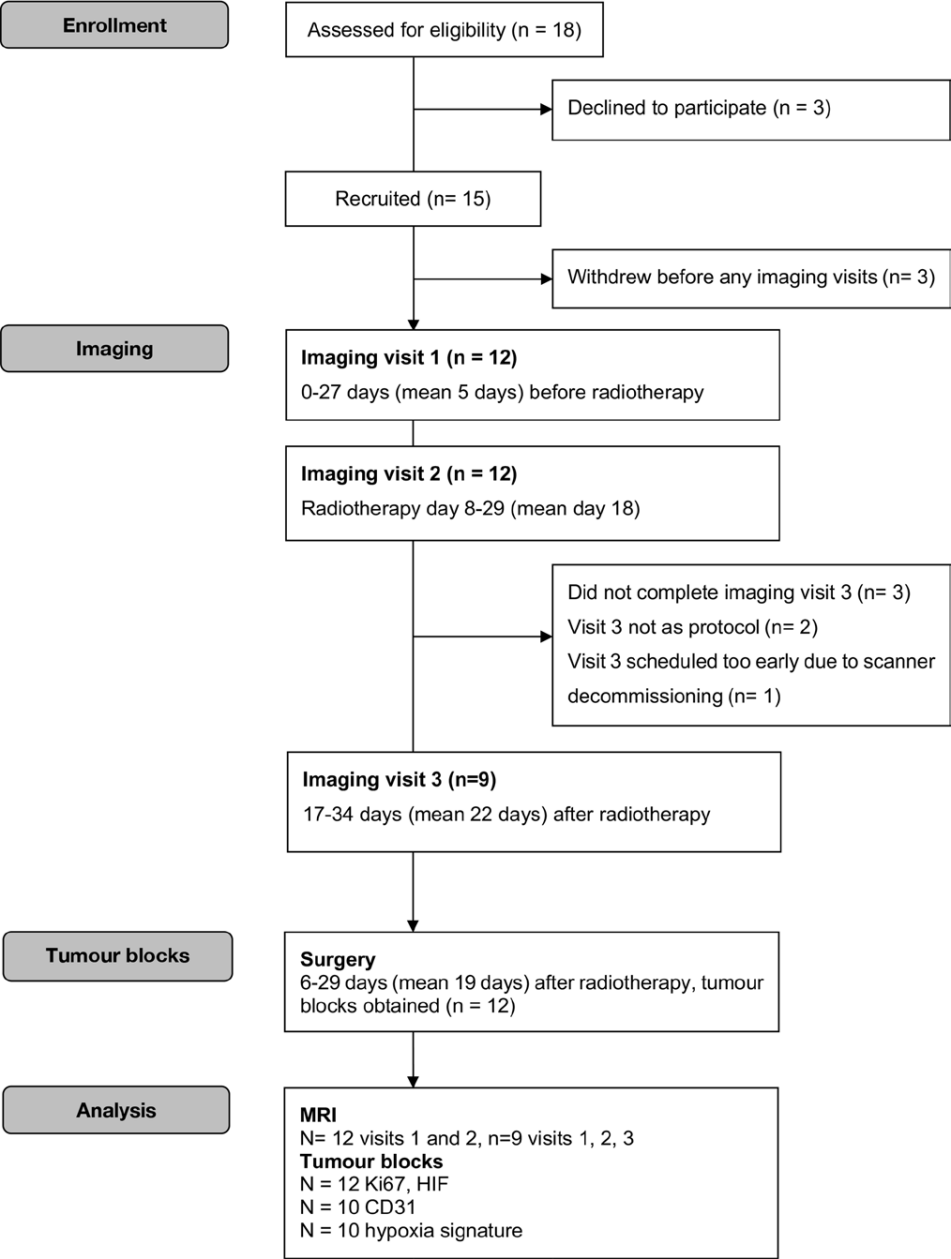
Modified CONSORT diagram.

Figure 1 shows a flow diagram for enrolment, imaging and analysis. Briefly, by the close of the study 15 patients were recruited and 12 scanned (9 had three scans, 3 had only the first two). All collected data were included in our analysis. As of January 2019, one patient had died from acute myeloid leukaemia and two had developed metastatic disease (one patient had a lung metastasis resected and the other developed a solitary spinal metastasis).

### Image analysis

**Figure 2.**
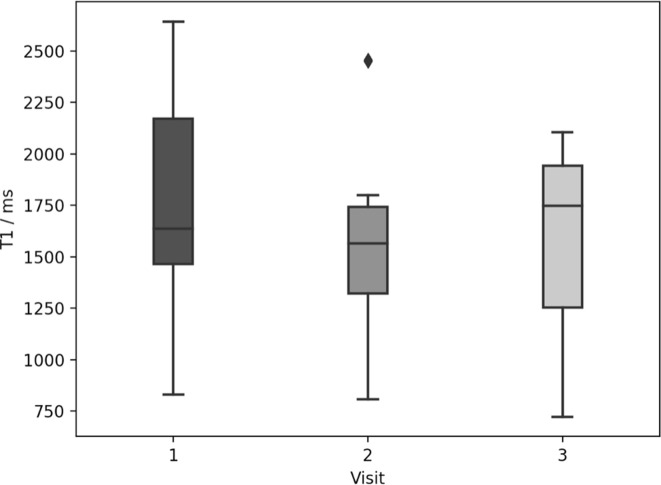
Box plot showing median T1 (box middle line), lower and upper quartiles (box edges) and data range (whiskers) over all patients for three visits. Outliers are shown as diamonds. There was a significant difference between median T1 at visit 2 compared with visit 1 (Wilcoxon signed ranks test, p=0.008). No other significant differences were detected.

**Figure 3.**
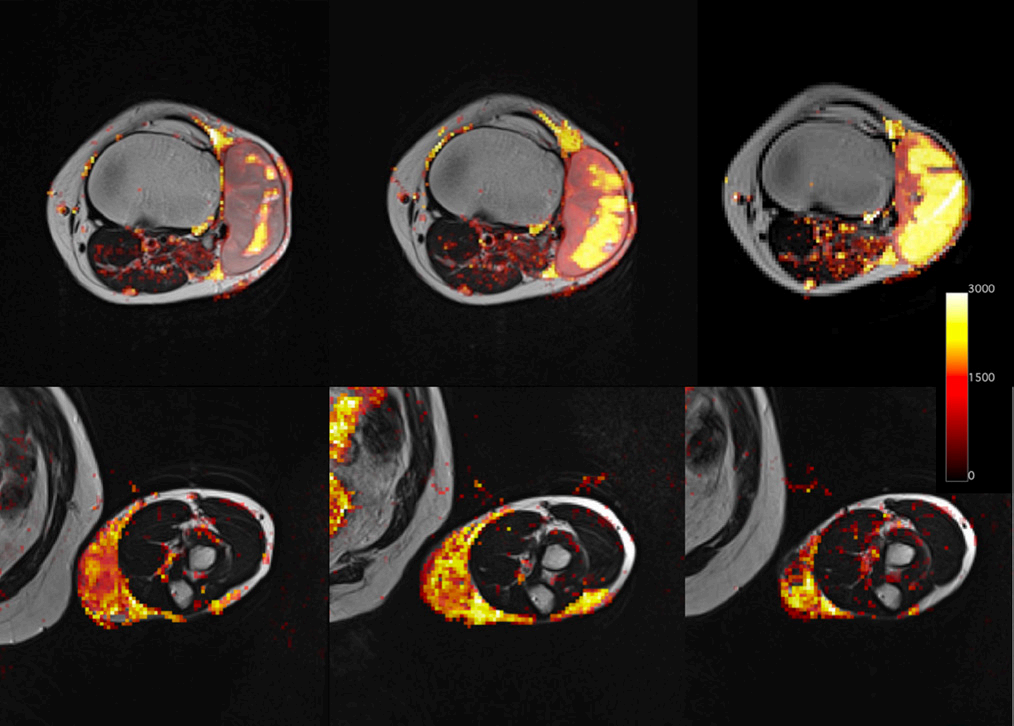
Example ADC (apparent diffusion coefficient, x10^-6^ mm^2^/s) maps superimposed on anatomical T_2_w images over three visits (left – before radiotherapy, centre – during radiotherapy, right – after radiotherapy) for two patients (Upper panel, lower panel).

No significant reduction in volume was observed across visits. The median volumes with their interquartile ranges in cm^3^ were: 29 (22–51) for visit 1, 34 (23–48) for visit 2 and 25 (10–32) for visit 3. In comparison with pre-radiotherapy values, there was a significant reduction in T1 at visit 2 (*p* = 0.008) (Figure 2) and in ADC at visit 3 (*p* = 0.04), with example ADC maps shown in Figure 3 for two patients. No significant changes in iAUC_60_ were seen over the three visits.

### Histological results

Pearson correlation coefficients between imaging and histological parameters are shown with their *p*-values in Table 3. Significant correlations were found between hypoxia scores and pre-treatment iAUC_60_ (*r* = −0.64, *p* = 0.03), pre-treatment ADC (*r* = 0.63, *p* = 0.03) and mid-treatment iAUC_60_ (*r* = −0.63, *p* = 0.03). There was also a significant correlation between mid-treatment ADC and Ki67 (*r* = −0.66, *p* = 0.02). Stratification of patients by CAIX staining demonstrated significant differences in T1 (visit 1 p = 0.003, visit 2 p = 0.01, visit 3 p = 0.03), and iAUC_60_ at visit 1 (*p* = 0.03).

**Table 3.** Pearson correlation coefficients for correlations between imaging and histological parameters., with *p*-values calculated from a *t*-distribution shown in brackets

	T1	iAUC	ADC	Volume
	Pre-treatment			
CD31	−0.07 (0.83)	−0.28 (0.37)	−0.16 (0.63)	−0.37 (0.23)
Ki67	−0.50 (0.10)	0.11 (0.74)	−0.55 (0.06)	−0.29 (0.36)
HIF	0.08 (0.80)	−0.40 (0.19)	0.04 (0.91)	−0.10 (0.76)
Hypoxia score	0.47 (0.12)	−0.64 (0.03)*	0.63 (0.03)*	−0.16 (0.63)
	Mid-treatment			
CD31	−0.09 (0.78)	−0.43 (0.17)	−0.31 (0.32)	−0.32 (0.31)
Ki67	−0.43 (0.16)	−0.05 (0.88)	−0.66 (0.02)*	−0.31 (0.33)
HIF	0.03 (0.94)	−0.44 (0.16)	−0.10 (0.75)	−0.13 (0.68)
Hypoxia score	0.24 (0.45)	−0.63 (0.03)*	0.58 (0.05)	−0.14 (0.66)
	Post-treatment			
CD31	−0.15 (0.63)	−0.26 (0.42)	−0.29 (0.36)	−0.13 (0.69)
Ki67	−0.57 (0.05)	−0.09 (0.78)	−0.44 (0.16)	−0.04 (0.66)
HIF	−0.27 (0.40)	−0.20 (0.53)	−0.36 (0.25)	−0.29 (0.37)
Hypoxia score	0.23 (0.43)	−0.27 (0.40)	0.34 (0.27)	−0.18 (0.57)

ADC, apparent diffusion coefficient; AUC, area under the curve; HIF, hypoxia inducible factor.

### Discussion

In this work, we developed a mpMRI protocol for STS, including established functional and structural imaging, that was acceptable for patients and that could be applied several times during radiotherapy. The imaging protocol is deliverable as shown by the good patient compliance, and there are some interesting findings. To our knowledge no other study has reported the use of mpMRI in STS in neoadjuvant radiotherapy with a time point early in treatment..

We applied the techniques of DCE-MRI, DWI and T1 measurement to explore radiotherapy-related changes to tumour tissue not reflected by changes in size. As found in several previous publications,^3,4,10^ size change varied between tumours and was not related to any histological parameters measured at surgery. Since conventional RECIST criteria cannot be associated with radiotherapy response, a non-invasive imaging biomarker predictive of overall survival, local control^7^ and distant metastasis^8,9^ is desirable and could be used to stratify patients for treatment intensification or de-intensification. The tissue T1 decreased significantly between baseline and mid-treatment but by the end of radiotherapy the main variation in T1 was between patients. T1 changes can reflect a wide range of alterations in tissue structure^26^ resulting in large variations in values between patients, which complicates interpretation of tumour revascularisation between baseline and early treatment. Tumours with positive staining for CAIX had a significantly shorter T1 at all three visits, consistent with the expected T1 shortening effect of deoxyhaemoglobin. ADC increased significantly between baseline and post-treatment, as shown in previous work^27^ and is hypothesised to reflect decreased cellularity and increased necrosis.^28^ Pre-treatment ADC values were similar to those reported in previous studies^27,28^ though, as noted by other authors, the range of baseline values was large.

There was an inverse correlation between iAUC_60_ and hypoxia score. iAUC_60_ is a semi-quantitative parameter with no direct physiological interpretation. A source of the negative correlation observed both at baseline and early in treatment could be poor tumour perfusion resulting in a lower iAUC_60_ and the post-treatment hypoxia observed. This is consistent with previous work in a mouse xenograft model, which showed reduced AUC in hypoxic tumour regions defined by pimonidazole staining.^29^ At baseline, tumours with positive staining for post-treatment CAIX had significantly higher iAUC_60_, which is not consistent with the expected relationship between iAUC_60_, perfusion and oxygenation. The inverse correlation between mid-treatment ADC and Ki67 at resection suggests that tumours with a good initial response to radiotherapy (lower cell density, higher ADC) subsequently have less proliferation at resection. The relationship between ADC and Ki67 has been explored previously, and reported in a meta-analysis that confirmed this negative correlation in many tumour types.^30^ Similarly, tumours that show no staining for CAIX at resection (normoxic at surgery) have a significantly higher ADC early in treatment (lower cell density). In a previous study in melanoma xenografts, ADC was shown to be inversely related to hypoxic fraction determined by pimonidazole staining.^31^ The relationship between CAIX and ADC has been explored previously but no relationship was found,^32^ possibly because CAIX is a downstream marker of hypoxia which can be regulated by other factors whereas pimonidazole represents a more direct measure. Correlation between pre-treatment ADC and hypoxia score is more difficult to interpret as ADC is measured long before the resection of the tumour.

This study has several limitations. The number of patients was small, prohibiting examination of differences between responders and non-responders. The results should be interpreted with caution due to sample size and differences in measurement timepoint, but the main aim of the study was to develop a suitable imaging protocol for which this small number is likely to be sufficient. The DCE-MRI data were acquired with sufficient temporal resolution to support tracer kinetics modelling, but the varying tumour locations made measurement of an arterial input function extremely challenging. We therefore opted to use a semi-quantitative parameter instead, but modelling could perhaps have given further insight.^17^ Future work could include modelling, if suitable spatial resolution can be obtained, and an MR-linac could allow more detailed monitoring during treatment.^33^

Overall, this work has resulted in a feasible imaging protocol aligning with domain I of the imaging biomarker road map. We identified significant changes in T1 and ADC during treatment. As iAUC relates to hypoxia, an established adverse prognostic factor in STS,^34^ it may be suitable as a non-invasive biomarker of tumour microenvironment and should be explored in a larger study.
